# Design and Implementation of Universal Cyber-Physical Model for Testing Logistic Control Algorithms of Production Line’s Digital Twin by Using Color Sensor

**DOI:** 10.3390/s21051842

**Published:** 2021-03-06

**Authors:** Ján Vachálek, Dana Šišmišová, Pavol Vašek, Ivan Fiťka, Juraj Slovák, Matej Šimovec

**Affiliations:** 1 Faculty of Mechanical Engineering, Slovak University of Technology in Bratislava, Námestie slobody 17, 812 31 Bratislava, Slovakia; ivan.fitka@stuba.sk (I.F.); juraj.slovak@stuba.sk (J.S.); matej.simovec@stuba.sk (M.Š.); 2 SOVA Digital a.s., Bojnická 3, 831 04 Bratislava, Slovakia; pavol.vasek@sova.sk

**Keywords:** cyber-physical model, digital twin, logistic control algorithms, color sensor, assembly lines, robotic workplace

## Abstract

This paper deals with the design and implementation of a universal cyber-physical model capable of simulating any production process in order to optimize its logistics systems. The basic idea is the direct possibility of testing and debugging advanced logistics algorithms using a digital twin outside the production line. Since the digital twin requires a physical connection to a real line for its operation, this connection is substituted by a modular cyber-physical system (CPS), which replicates the same physical inputs and outputs as a real production line. Especially in fully functional production facilities, there is a trend towards optimizing logistics systems in order to increase efficiency and reduce idle time. Virtualization techniques in the form of a digital twin are standardly used for this purpose. The possibility of an initial test of the physical implementation of proposed optimization changes before they are fully implemented into operation is a pragmatic question that still resonates on the production side. Such concerns are justified because the proposed changes in the optimization of production logistics based on simulations from a digital twin tend to be initially costly and affect the existing functional production infrastructure. Therefore, we created a universal CPS based on requirements from our cooperating manufacturing companies. The model fully physically reproduces the real conditions of simulated production and verifies in advance the quality of proposed optimization changes virtually by the digital twin. Optimization costs are also significantly reduced, as it is not necessary to verify the optimization impact directly in production, but only in the physical model. To demonstrate the versatility of deployment, we chose a configuration simulating a robotic assembly workplace and its logistics.

## 1. Introduction

We have many options nowadays for the optimization of existing or future logistics production processes in order to increase their competitiveness. The concepts of intelligent industry following Industry 4.0 standards allow us to fully virtualize and digitize industrial production processes for optimization purposes. The term Industry 4.0, known as the Fourth Industrial Revolution, was first introduced at the World Industrial Technology Fair in Hanover in 2011 [1]. Based on this concept, in 2014 the German government declared Industry 4.0 to be a key part of their “High-Tech Strategy 2020 for Germany” initiative. Based on this strategy, other governments and global industry leaders [2] have gradually embraced the strategy worldwide as something fundamental to their future direction. The strategy is based on the complete digitization of industry in the form of information and communication technology (ICT) deployment with automation in the form of cyber-physical systems (CPS) using the Internet of Things (IoT) and Internet of Services (IoS) [3]. Their role is consequently optimized to produce effects deep in the production process, with additional possibilities and variations unimaginable so far, as stated in the referenced literature [4,5,6,7].

The current state-of-the-art regarding Industry 4.0 is very extensive and still evolving rapidly. The basic features of Industry 4.0 can be summarized in four basic areas that lead to the establishment of intelligent factories [8].

The first area is the vertical interconnection of intelligent production systems, such as intelligent factories and intelligent products. For example, the interconnection of intelligent logistics, production, marketing and intelligent services with a strong focus on individual customer needs. The second area is horizontal integration through the new generation of value-added global information networks, including the integration of business partners and customers, new business models and cooperation across countries and continents. Independently of these areas, there is the third area, which focuses on the application of technology throughout the value chain, not only in the production process but also in the final product, thus covering the entire product life cycle. The fourth area is the exponential deployment of technologies. Although these technologies may not be new, they are becoming capable of mass application because their performance is heavily increasing and their prices are rapidly decreasing (e.g., simple sensors). The idea of an intelligent factory can be defined in terms of Industry 4.0 concepts by applying the above areas. Theoretically, it is based on nine technological pillars: the industrial Internet of Things (IoT) in the form of CPS, big data and analytics, product lifecycle management (PLM) systems, digital manufacturing, cloud computing, augmented reality, autonomous or collaborative robots, additive manufacturing and cybersecurity [9,10,11,12,13,14,15,16,17,18,19,20,21].

We focus in this paper on digital manufacturing and IoT, which allow us to optimize existing or planned production lines using a digital twin. We could define a digital twin as a virtual representation of a physical product or process. The digital twin is able to demonstrate the impact of diverse process changes on virtual models and predict their impact without the physical implementation of planned changes. In this way, it reduces the costs and downtime of the process when it is not necessary to implement every proposed scenario. Instead, only the optimal solutions are deployed. In our case, we use a digital twin to control the logistics process. For the proper functionality of the digital twin, unification with real production is necessary. The connection between physical and virtual worlds comes from the identification of manufactured components using a color sensor. Sensor data are useless if the digital twin does not fully replicate the behavior of the real system. It is possible to precisely tailor the solution when creating a digital twin for a specific, relatively constant system. However, one of our goals is to create a universal system, applicable to any production, wherein suitable data collection is ensured. The system must create a structure based on the input data to replicate the real system. This process takes place in the initialization phase. A virtual digital twin is automatically generated without external intervention based on the input data from database tables. Similarly, based on the same input data used by digital twin CPS, we create a physical model that represents the required physical inputs and outputs. The connected CPS fully replaces the need to verify optimization logistics processes directly in production. Therefore, it has a significant impact on the speed of development of optimization algorithms via the digital twin and leads to lower-cost solutions.

We introduce the concept of connecting a digital twin with a cyber-physical system. The previous approaches considered either only optimization using a digital twin or the possibility of deploying CPS systems into production. The cooperation of a CPS and a digital twin in verifying the functionality of the optimization from the digital twin as described in this paper has not yet been presented [22,23,24,25,26,27,28,29,30,31,32].

## 2. Methods and Materials

**Figure 1:**
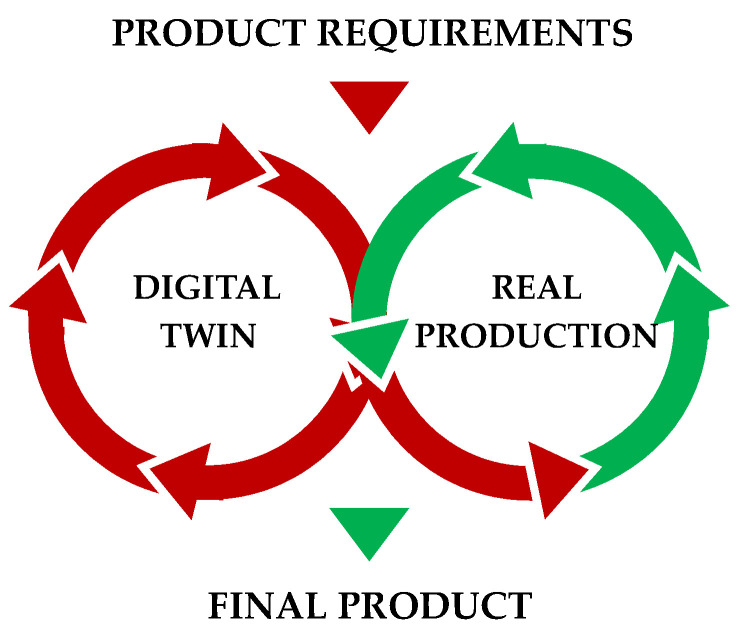
Digital twin visualization.

The number of possibilities is sometimes an obstacle in conservative industrial production. New opportunities are offered by optimization processes, which nowadays bring the possibility of saving energy, shortening production times, minimizing idle time and thus reducing production costs. However, these changes come with obstacles. Especially with existing production lines, it is necessary to thoroughly analyze all of the internal processes and production operations, which can lead to a temporary increase in the number of employees. Sometimes it is also necessary to take a more radical approach, i.e., to test partial changes directly in production. For this reason, we developed and built a real cyber-physical third-generation system, capable of storing, analyzing and processing larger data sets, as mentioned in [33,34,35]. This suggestion has been initiated based on our presentation of optimizations performed in the form of digital twin output animations and calculations in the Siemens Tecnomatix Plant Simulate environment. The most common existing operation issues are production micro-idle times and the efficient logistics of individual workplaces. To avoid these issues, it is necessary to identify them and then subsequently select and test an optimization algorithm suitable for flexible production such as the milk run, ant colony or Kanban algorithm [36,37,38,39,40,41,42,43,44,45,46,47]. The best option for testing is a virtual environment with a digital twin that is directly connected to physical sensors in production, in accordance with the methodology shown in Figure 1.

Based on this, we decided to build the model and set the following basic conditions: The model must be universal and able to simulate any real production line. It must be portable, because the presentation of Industry 4.0 benefits usually takes place at the client site, e.g., in the factory. It must be simple, modular and easily expandable by new workplaces. Its price must be as low as possible, but not at the expense of quality. A physical model built in this way fully replaces the need to test optimization algorithms directly in production. It is highly modular because the number of workplaces depends only on consecutive connected stations, the number of which is unlimited. Based on the initial parameters, it can represent any physical sensors in the production environment as required by the virtual digital twin environment.

In addition to the basic requirements mentioned above, we had to solve the basic problem of placing sensors on the model, which has a major impact on the accuracy of simulations from the physical model. As this is an essential theoretical consideration, we address this in more detail below.

While controlling a logistics system, it is necessary to receive information in advance from workplaces about the amount of consumed material. Based on this information, the control system can evaluate the need for an order for additional materials in the workplace and assign priority to it. A key factor influencing early information acquisition is the location of sensors in the production process (e.g., see [48]).

**Figure 2:**
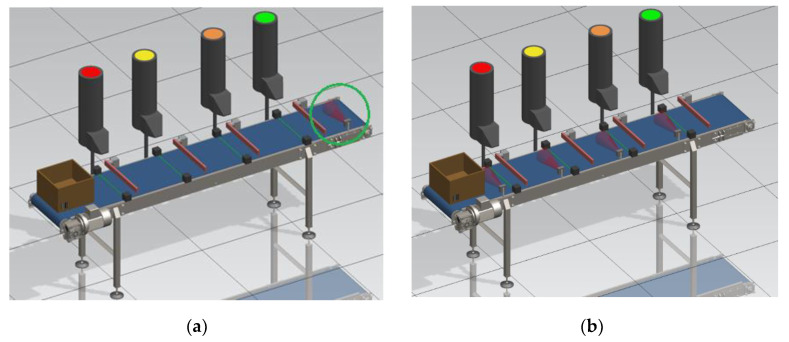
Sensor position: (**a**) at the output of the line; (**b**) at each workplace separately.

If workplaces in the production process are separated, the simplest way is to place sensors in each workplace. This ensures that current data are retrieved after each operation at the workplace. However, if the workplaces are located one behind the other, e.g., along a conveyor belt, we have several options for positioning the sensors. The first option is to place one sensor at the exit of the conveyor belt, as shown in Figure 2a.

Figure 2a shows a simplified model of a production line for filling a box moving along a conveyor belt, which we created in the Siemens Tecnomatix Process Simulate software (Siemens PLM Software, Plano, TX, USA). There are four color-coded workplaces on the line, each consuming the material that is filled into the box. At the output of the line is a sensor that identifies the box. The advantages of using one sensor at the output of the line are a reduction in the technical complexity of implementation, a reduction in the amount of transmitted data and lower costs for implementation.

One disadvantage of this configuration is the timeliness of the acquired data. If there are a large number of workplaces on the line and there are a number of products in the production process at the same time, information from the line output may not be sufficient to control the logistics system. Until the first product reaches the line output, the first workplace may be out of material. Therefore, the use of this configuration is more suitable for lines with a lower number of workplaces. Another disadvantage of this configuration is the required recording of failures or products removed from the line earlier, e.g., due to quality testing. If the box was filled at the first three workplaces and removed from the process before the fourth workplace, it would not be recorded at the output, even though there was consumed material at the first three workplaces. This material would be missing only in the real system because the digital control system would only deduct materials from the count based on the signal from the sensor at the end of the line, thus creating inaccuracies between the real and digital systems. When implementing this configuration, it is necessary to create a database for recording products excluded from the process and updating the state of material containers at workplaces.

An alternative configuration is to place sensors in multiple positions along the line or to place a sensor at each workplace, as shown in Figure 2b. By placing a sensor at each workplace, we gain more control over the processes on the line. On the other hand, this option requires the use of more hardware parts and also results in a higher amount of transferred data, as shown in [49]. The configuration of sensor locations, therefore, depends on the specific process in which the sensors will be applied. To test individual configurations, communication, control algorithms, transmission and data collection from a process, we created physical and simulation models which we describe in the following sections.

## 3. Functional Description of a Physical Model Workplace

Each physical model workplace represents certain production operations required for the final product’s completion. In this section, we describe how the individual workplaces operate. After enabling one workplace, the initialization phase begins. In the initialization phase, the workplace downloads a bill of material (BOM) for simulated production based on its unique identifier. The BOM for each workplace defines how many pieces of which material type are consumed if the product’s color is identified. As this is a flexible production process representation, each defined color can consume different amounts of various materials. After downloading BOM, the workplace also downloads a matrix containing the initial quantity of particular materials available at the workplace. After initialization, the workplace is activated. Since the individual workplaces are connected in parallel, it is also possible to simulate failures at specific workplaces.

When a component arrives at the workplace, it is scanned by a photogate. The photogate switches a timer, which, based on the belt speed, initiates the gate and thus stops the component. When the component stops, it is detected by the color sensor. At this point, two possibilities can occur; i.e., the sensor either identifies the component or does not identify the component. If the component is not identified, the gate is opened and the component leaves the conveyor belt. Any operation is performed for the component and the error is recorded into the database. If the component is identified, then component arrival time; a unique identifier assigned by the system; and measured R, G and B values are recorded into the database.

**Figure 3:**
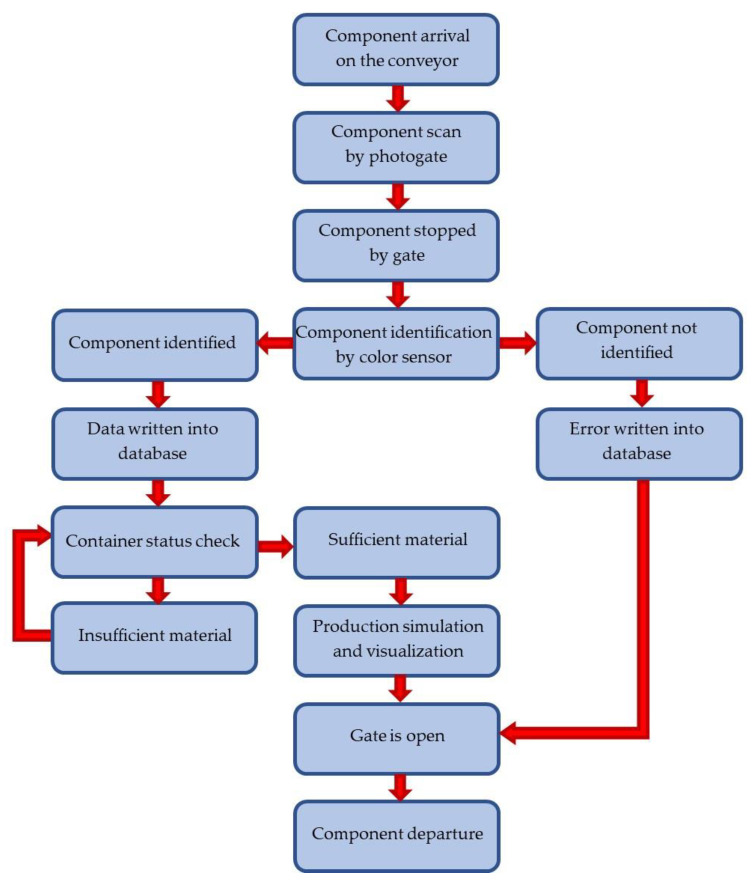
Workplace operations flowchart.

In the next step, the amount of required material and the duration of these workplace operations are determined from the database data. If there is an inadequate quantity of products in the virtual container, the workplace waits for material refill. If there is a sufficient amount of material, production is simulated. After simulation completion, the gate opens and the component continues along the conveyor belt to the next workplace. The operations of workplaces are shown in the flowchart in Figure 3.

## 4. Design of a Physical (Real) Model of a Cyber-Physical System for Testing Control Algorithms

The modular workplaces were designed on the embedded Wemos platform with two additional color display modules. We connected individual workplaces with a conveyor belt. The number of workplaces is theoretically unlimited and depends only on the belt length and production requirements. We created a universal programming environment in Python with a MySQL database connection. We stored the parameter of the individual workplace (in our case a robotic workplace) in the database, but in general it can be stored for any workplace. The basic parameters of the workplaces are saved in the database for future use. We created various combinations of colored rectangles to keep modularity of products on the line. Thus, all available products produced on the model production line can be detected and simulated based on their color. From a logistic perspective, simulated products need various material supplies that are ideal for their optimization. The product waits at each workplace for a certain amount of time, the same as during real production (or the presented optimization simulation) for its processing or completion. The individual phases of processes are graphically shown on the display together with the state of material containers. A strong emphasis is put on visualization and clarity of information.

The target was to make outputs from a digital twin and a physical model as similar as possible for the sake of imagination. We met this target and, based on results and measured optimization times, confirmed that the model accurately correlated with simulated real operation and subsequently with simulated optimization via the digital twin. This was a crucial moment necessary for the credibility of the model in the eyes of the stakeholders to whom this optimization method was presented.

**Figure 4:**
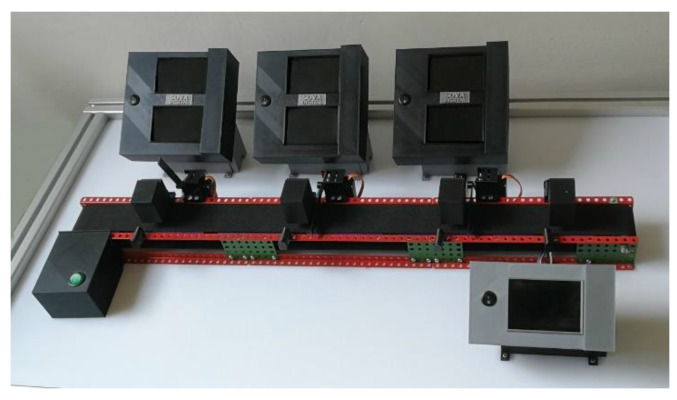
The final physical model.

The physical model is used to test the connection of the created logistics system to real hardware and collect data for different functional test scenarios for the logistics system. The priority for the physical model design was to make it compact, modular, lightweight, portable and as easy to connect as possible. Another requirement was the low price for individual simulation workplaces, so that we could extend the model if necessary. In accordance with these assumptions, we created a base for the model from aluminum profiles with inserted plexiglass. The model is shown in Figure 4.

There is a production line placed on the base with three workplaces simulating various production operations. The types of simulated production operations and the number of workplaces are practically unlimited. In our case, we have designed three workplaces, but since this is a universal concept, their number can be increased or decreased as requested. For the definition of a specific production operation, we need to know its duration. A unique identifier is assigned to each operation in the database of workplaces. We also need to know which materials and components are required to perform given manufacturing operations in order to provide logistics. Finally, the sensors are assigned to an operation so that we can synchronize the digital twin. In our case, the output is the fourth (last) workplace and serves to identify the product by a single sensor configuration. We designed and created by 3D printing all workplace parts except the conveyor belt. In front of each workplace, there is an optical gate that indicates an approaching component. The optical gate consists of an infrared photodiode and photoresistor placed opposite each other. This gate sends a signal when the light beam is crossed by the product. The signal triggers a servomotor, which closes the gate and thus stops the product.

Each of these simulated workplaces contains a virtual container of parts that are consumed by the production. As this is a flexible production, the amount of components consumed depends on the final produced product. Therefore, the product must first be identified, and it must then be determined which production operations will be performed. Variability is important for flexible production. Due to that, three types of sensors were taken into consideration for product identification.

### 4.1. Product Identification

The first choice when selecting sensors for product identification was radio frequency identification device (RFID) sensors, which are very often used in industry. An RFID chip mounted on the product would be used for identification. The advantage of RFID is its high resolution distance, reading speed and especially the possibility of reading without direct visibility of the chip. However, this method was not applicable for our model due to its dimensions. As the width of the conveyor belt is 5 cm, the size of the products is adapted to 4.5 × 4.5 cm. So-called weak spots occur when stacking products, due to interference from chip transmitters, and cause errors in reading data; such errors are solved, e.g., in [50].

The second option was a barcode reader for stickers or printed barcodes on the product. The advantage of this method compared to RFID is the possibility of stacking products; therefore, it would be suitable for our model. However, due to the dimensions of barcode readers and compatibility with used hardware, we decided to use a color sensor to identify the products at the experimental workplace.

#### Color Sensor TCS 230

TCS 230 (Texas Advanced Optoelectronic Solutions Inc., Plano, TX, USA) is a programmable sensor that converts light intensity to frequency. The sensor consists of configurable silicon photodiodes and current–frequency converters placed onto an integrated Complementary Metal Oxide Semiconductor (CMOS) circuit. Digital inputs and outputs allow a direct connection to a microcomputer or other logic circuits. The converter reads values from 64 connected 120 × 120 μm photodiodes, where 16 photodiodes have a blue filter, 16 have a green filter, 16 have a red filter and 16 have no filter. The integration of four photodiode types minimizes the impact of irregularities. Photodiode type is controlled by logic inputs S2 and S3. The output frequency scale is controlled by logic inputs S0 and S1 [51].

TCS 230 sensor proved to have less measurement accuracy and lower measuring distance compared to the standard industrial color sensor CSM-WP117A2P (SICK AG, Waldkirch, Germany), which is expected due to its low purchase price. It was possible to identify several colors with the used sensor, but the control limits were considerably wider compared to the industry-standard sensor CSM-WP117A2P. Measurement was always performed when the component was stopped. TCS 230 sensor was sufficient for the model purpose, but it is not suitable for industry usage. We will use sensor CSM-WP117A2P or a sensor with similar properties for the deployment of the logistics system. Potential issues with color detection and the influence of external parameters of the sensor CSM-WP117A2P are discussed in detail in [52].

After component identification, the measured data are recorded into the database and the simulated production process begins. The progress of this process is shown on the display representing the container.

### 4.2. Process Visualization

**Figure 5:**
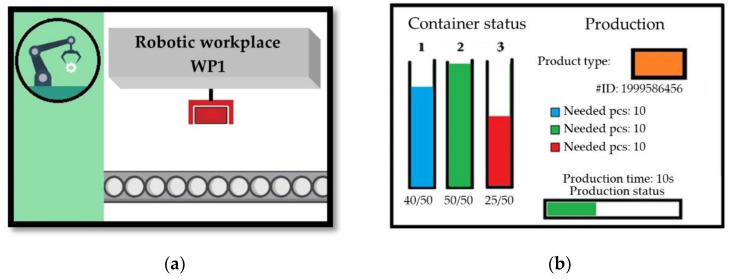
Robotic workplace visualization: (**a**) home screen; (**b**) display of production status on TFT display.

There are two 3.5-inch color Thin Film Transistor (TFT) display modules (Shenzhen QDtech Co., Ltd., Shenzhen City, Guangdong, China) with a resolution of 480 × 320 pixels at each workplace. The first display indicates the current activity at the given workplace. An example display of a workplace during the production process is shown in Figure 5.

Figure 5b shows a visualization of a simulated robotic assembly workplace. Upon a component’s approach, an animation starts, representing the steps performed by the robot. The second display represents the workplace container and shows ongoing process data. The left side of the display shows the current status of the material container for the given workplace, which is frequently read from the database. On the right side of the display, there is information about current activity at the workplace. Product type is displayed after component identification, based on the color. The product identification number is displayed below the product type. The product identification number is a unique number assigned to each product, based on which each product can be identified in the database. Below that is displayed the necessary materials needed to complete the production operation. This is read from the database together with duration of the production. As production is simulated, production status is indicated by a progress bar at the bottom of the screen.

### 4.3. Physical Model Control

**Figure 6:**
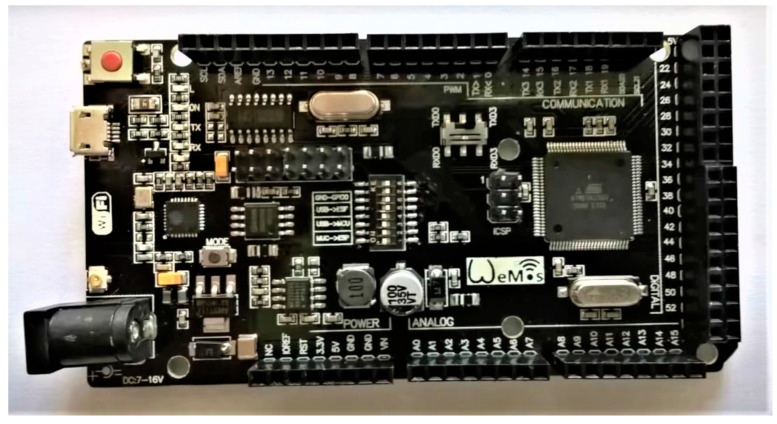
Wemos Mega development board.

The physical model is driven and sensors are controlled by a Wemos Mega development board (Kuongshun Electronic Limited, Shenzen, China) with an 8-bit ATmega 2560 microcomputer (Microchip Technology Inc., Chandler, AZ, USA). There are 54 digital input/output pins on the board. Pulse-width modulation is possible for 15 of them. Apart from these, 16 analog inputs and 4 hardware serial links are available on the board. This development board can be connected to a computer via its built-in micro-USB port using the converter between USB and the serial line mentioned in [53]. The board is shown in Figure 6.

The main advantage of the Wemos Mega board compared to the classic Arduino Mega 2560 (Zhongshan Baijia Dagu Electronic Technology Co., Ltd., Guangdong, China) is its integrated Wi-Fi module ESP8266, which was also used in [54]. Communication between the physical model and the database is ensured through this module. ESP8260 can work in web server mode, but for our needs, it will connect as a client to a database server based on [55]. This module with integrated TCP/IP protocol has a separate 32-bit processor and 4 MB of memory; therefore, it can operate independently from microcomputer.

## 5. Discussion

The aim of this paper was to present the concept of connecting a digital twin with a real physical cyber-physical system (CPS), which fully replaces the need for testing in real production. A similar approach was missing in practice. Based on these requirements, a CPS system was created that physically models a real production line. A complex software environment that can automatically generate digital twins using universal objects was built. For optimization purposes, the digital twin needs real physical inputs from the sensors in production, based on which a selected algorithm is used to recalculate the optimal supply of workplaces. Workplaces respond to improved logistics by increasing productivity and reducing energy consumption and production costs. The solution was also experimentally verified when we performed the optimization of a robotic assembly workplace. We used six different color combinations to simulate six different products, as flexible production with different product variations is currently preferred. Each product was assigned different material consumption configurations and different production times. Finally, we verified the capability and performance of an automatically generated digital twin and created the CPS. Verification came from a comparison of the final states of tables for produced products. The remaining materials and failures at the workplaces were also compared between digital twin and CPS, as they were kept separate from each other. The tables were identical, which confirmed the absolute match between the operations performed by the digital twin and the CPS.

We plan to expand the possibilities of optimizing logistics with the Kanban system [40] and ant colony [47] algorithms compared to the currently used milk run algorithm [42] in the future. We will also consider the analysis of historical data using machine learning algorithms to refine estimates of future material consumption and their better availability in warehouses.

We can state that the approach presented in the paper allows us to fully optimize the logistics processes of real production workplaces without any physical presence at the production site. This approach speeds up the optimization process and reduces its costs, as there is no need to partially verify optimization in real production. The concept is universal and allows the physical replacement of any production workplace with different degrees of complexity. The self-generating digital twin virtual model is also an innovation to increase productivity. The creation of a modular physical model based on the CPS system capable of storing, analyzing, processing and communicating is a typical example of modern CPS system deployment. The synergistic system built in this way is ultimately beneficial for both industry and research.

## Data Availability

Not applicable.
